# Identification of group specific motifs in Beta-lactamase family of proteins

**DOI:** 10.1186/1423-0127-16-109

**Published:** 2009-12-03

**Authors:** Reema Singh, Akansha Saxena, Harpreet Singh

**Affiliations:** 1 Biomedical Informatics Center, Indian Council of Medical Research, New Delhi, India

## Abstract

**Background:**

Beta-lactamases are one of the most serious threats to public health. In order to combat this threat we need to study the molecular and functional diversity of these enzymes and identify signatures specific to these enzymes. These signatures will enable us to develop inhibitors and diagnostic probes specific to lactamases. The existing classification of beta-lactamases was developed nearly 30 years ago when few lactamases were available. DLact database contain more than 2000 beta-lactamase, which can be used to study the molecular diversity and to identify signatures specific to this family.

**Methods:**

A set of 2020 beta-lactamase proteins available in the DLact database http://59.160.102.202/DLact were classified using graph-based clustering of Best Bi-Directional Hits. Non-redundant (> 90 percent identical) protein sequences from each group were aligned using T-Coffee and annotated using information available in literature. Motifs specific to each group were predicted using PRATT program.

**Results:**

The graph-based classification of beta-lactamase proteins resulted in the formation of six groups (Four major groups containing 191, 726, 774 and 73 proteins while two minor groups containing 50 and 8 proteins). Based on the information available in literature, we found that each of the four major groups correspond to the four classes proposed by Ambler. The two minor groups were novel and do not contain molecular signatures of beta-lactamase proteins reported in literature. The group-specific motifs showed high sensitivity (> 70%) and very high specificity (> 90%). The motifs from three groups (corresponding to class A, C and D) had a high level of conservation at DNA as well as protein level whereas the motifs from the fourth group (corresponding to class B) showed conservation at only protein level.

**Conclusion:**

The graph-based classification of beta-lactamase proteins corresponds with the classification proposed by Ambler, thus there is no need for formulating a new classification. However, further characterization of two small groups may require updating the existing classification scheme. Better sensitivity and specificity of group-specific motifs identified in this study, as compared to PROSITE motifs, and their proximity to the active site indicates that these motifs represents group-specific signature of beta-lactamases and can be further developed into diagnostics and therapeutics.

## Background

Beta lactamases are enzyme responsible for resistance to penicillin, cephalosporin and related beta lactam compounds. The enzymes hydrolyze the beta-lactam ring of these antibiotics and thus inactivate these drugs [1]. Almost as soon as a new beta-lactam antibiotic is introduced into the clinical usage, some previously unrecognized beta-lactamase with the capability of destroying this activity is identified [2], thus making beta-lactamases a serious threat to public health. In order to combat this threat we need to study the molecular and functional diversity of these enzymes and identify signatures specific to these enzymes. These signatures will enable us to develop inhibitors and diagnostic probes for the beta lactamase enzymes.

Beta lactamases show extensive molecular and functional diversity. Based on the characteristics of the enzymes and their substrate profile, a number of classification schemes have been proposed [3,4]. Among these, a functional classification scheme proposed by Ambler [5] is most widely accepted and used. In this scheme beta-lactamases have been divided into four classes i.e. A, B, C and D based upon their amino acid sequences [5]. Ambler originally specified two classes *i.e*. class A, the active site serine beta lactamases and class B the metallo-beta lactamases that require a bivalent metal ion, usually Zn^2+ ^for their activity. Later class C and class D were added to this classification. Enzymes from class A, C and D contain serine-based active site. Proteins from class A, C and D show sufficient structural similarity indicating that these may have descended from a common ancestor [6]. Class B consists of metallo beta lactamases and is perhaps the most heterogeneous class among all the classes of beta-lactamases. It has been further divided into a number of sub-classes [7]. In recent years, many new lactamases belonging to class B have been identified and sequenced. Their clinical importance is highlighted by the fact that these can hydrolyze carbapenems compounds which most often escape the activity of serine beta lactamase. The class B lactamases have been divided into three sub-classes B1, B2 and B3 [8].

Each class contains specific signature or motifs [1]. For example sequence belonging to class A contain three conserved elements *i.e*. S-X-X-K, S-D-N and K-T-G at positions 70, 130 and 234 respectively. Sequence belonging to class C contains S-X-S-K, Y-S-N and K-T-G at position 64, 150 and 314 respectively. Class D lactamase contains S-X-X-K, Y-G-N and K-T-G at positions 70, 144 and 214 respectively. Sequences belonging to class B contain H-90, D-92, L-117, H-168, G-204 and H-236 as conserved residues located at the bottom of the active site. Among these H-80, H-90 and H-168 accommodate Zn^2+ ^which is required for the activity of class B beta-lactamases [1].

However, the above mentioned classification and identified class-specific motifs are useful; these have been identified using a limited set of sequences. Recently we have developed a database of beta-lactamase genes, identified from sequenced bacterial genomes and plasmids [9]. This database contains 2020 beta-lactamase genes from 457 bacterial strains and offered us an opportunity to study diversity of lactamase genes and to identify molecular signatures of lactamase family. The classification approach used in this study is based on evolutionary relationship between beta-lactamase proteins and hence closer to natural classification. Group-specific signatures were also identified from sequences in each group.

## Methods

### Data

Protein and DNA sequences of 2020 lactamase genes available in DLact database [9] were used in this study. The lactamase sequences in DLact database were identified using experimentally identified lactamase proteins. The chromosomal and Plasmid DNA sequences of 457 sequenced bacterial strains were scanned for genes homologous to these experimentally identified lactamase proteins using BLASTx [10]. Each putative lactamase gene was annotated using Interproscan, rpsBLAST, Pfam and SMART. The database is available at http://59.160.102.202/DLact

### Classification of genes

**Figure 1:**
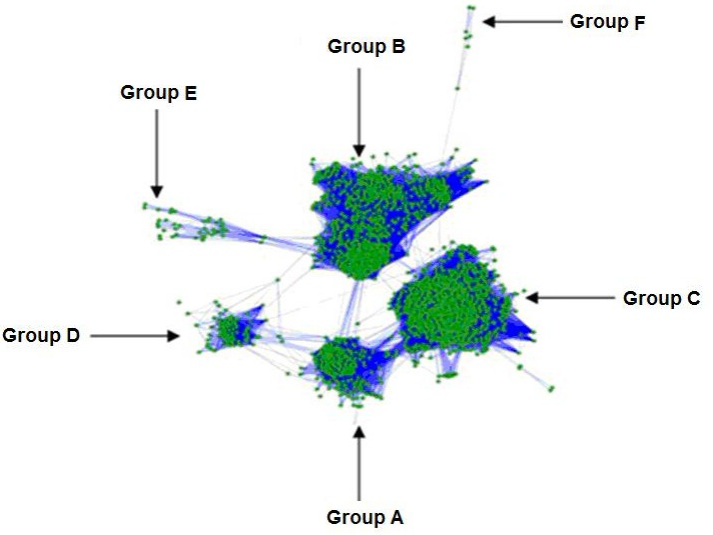
**Graph-based clustering of Bi-Directional Best hits of lactamase genes**. Six groups of the sequence can be seen. The labelling of four major groups is based on characteristics of class A, B, C, and D lactamases in literature.

The protein sequences of lactamase genes were separated according to their source genome. Each lactamase protein from a genome was compared to all proteins from another genome using BLASTP. Pair of sequences that were best hits when either sequence was used as query was identified as BeTs (Bi-directional hits), and the sequences in pair were considered as functionally related. BeTs relationship is one of the bare operational definitions of orthology. If the best hit was in only one direction, then no relationship was assumed between the sequence pair. BeTs pairs were clustered using a procedure adopted for developing COG (Cluster of orthologous groups) database [11]. In addition to above criterion, we imposed an e-value cutoff. Thus, a sequence pair was considered to be related by BeTs if in both direction, the e-value associated with each BLAST comparison was less than 0.001. Since in this approach only comparisons between proteins from two separate genomes were made, obvious paralogous were avoided. Furthermore, introduction of the e-value cutoff eliminated spurious matches. The output of this step was a graph with the lactamase genes as nodes and Best Bi-directional Hit relationship between a pair of genes as edges. This graph was used as an input to the cytoscape [12] which performed graph-based clustering of lactamase genes [Figure 1].

### Identification of group-specific motifs

Redundancy in sequence from each group was removed using CD-HIT [13] with a cut-off of 0.9. Motifs in each of the non-redundant set were searched using PRATT [14] at various 'C%' values. The parameter 'C%' signifies minimum percentage of sequences that should match a motif. Increase in 'C%' increases specificity of identified patterns. Group-specific sensitivity and specificity was calculated for each pattern as:

Where TP(True Positive) is the number of sequences from a selected group containing a given motif, FP(False Positive) is the number sequences from other groups containing given motif and FN(False Negative) is the number of sequences from selected group that do not contain given motif.

## Results

The graph-based classification of beta-lactamase proteins resulted in the formation of six highly interconnected groups (Figure 1). The protein sequences within each group showed dense BeTs relationships, while few relationships between sequences across the groups were also observed. We arbitrarily labelled these groups as Group A-F. Among these, four major groups (Group A-D) contain 191, 726, 774 and 73 proteins while two minor groups (Group E and F respectively) contain 50 and 8 proteins.

Based on the information available in literature about the molecular characteristics of beta-lactamase proteins [1], we found that each of the four major groups corresponds to the four groups proposed by Ambler [5]. Interestingly proteins from the group E and F does not contain beta lactamase domain or signatures specific to beta lactamases reported in literature. Proteins from both these groups showed BeTS relationship with proteins from Group B. Sequence from each the four major groups contain at least one conserved element, characteristic of beta lactamases [1]. The two minor groups do not contain molecular signatures of beta-lactamase proteins reported in literature. However, the density of BeTS relationships indicates that these are potentially novel types of lactamases or highly divergent relatives of Group B lactamases. These proteins are uncharacterized in NCBI. Further analysis is needed to characterize sequences from these groups.

**Figure 2:**
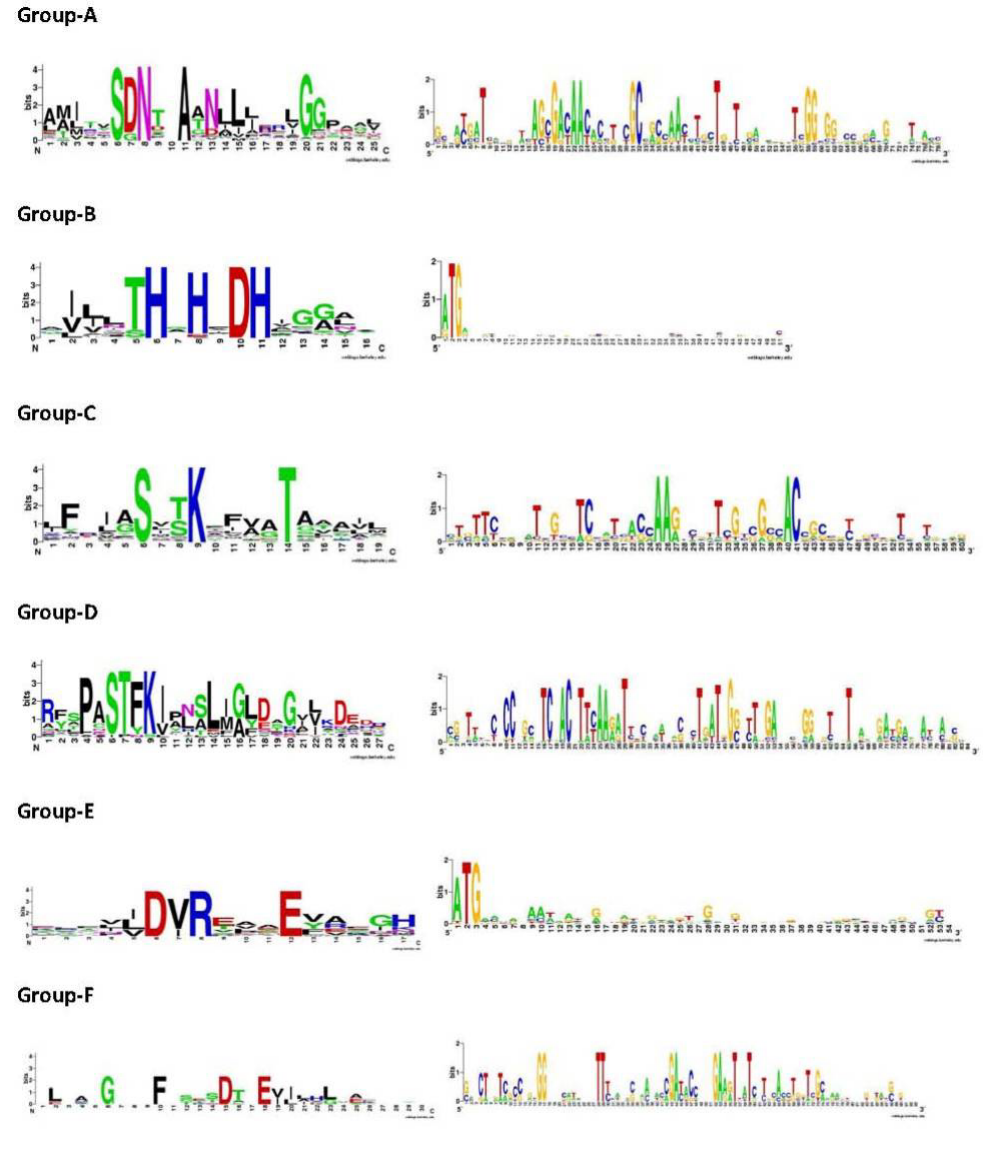
**Sequence logos of group-specific motifs identified in this study at protein and DNA level**. Except group B and E, there is significant conservation at proteins as well as DNA level.

Redundant sequences from each group were removed using CD-HIT and the non-redundant sequences from each group were aligned using T-Coffee [15]. The group-specific motifs identified by PRATT from each group had a high sensitivity (> 70%) and very high specificity (> 90%). The motifs from groups A, C and D (corresponding to class A, C and D of Ambler classification) showed a high level of conservation at DNA as well as protein levels (Figure 2). Sequence belongs to class E does not shows any sequence conservation. Whereas sequence belongs to class F contain G, P, N, F, D and E towards the N-terminal (Figure 2). The motifs identified from current study were compared to group-specific motifs present in the PROSITE [16] database. It was observed that PROSITE motifs were significantly less sensitive than motifs identified in present study. It is likely that the large number of sequences used in identification of motifs has resulted in higher sensitivity.

**Figure 3:**
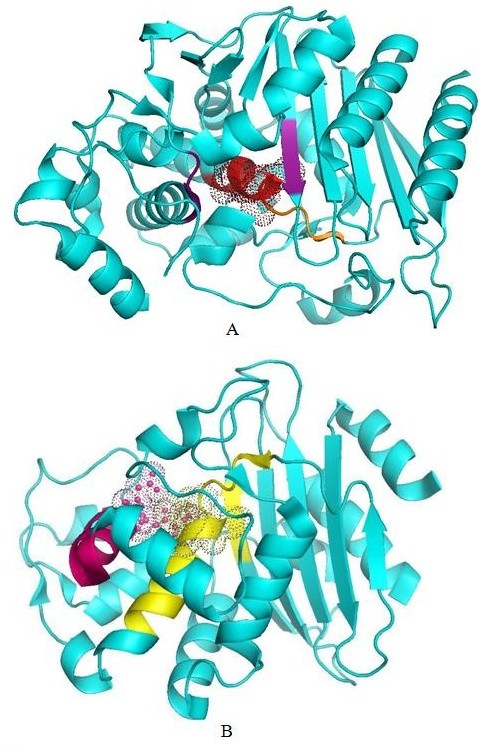
**A. Three dimensional model of class C protein **(PDB code 2QZ6) **showing group C specific motif identified from present study in red colour and class C specific PROSITE motif in orange**. The active site region is highlighted with dotted surface while the known two of the three conserved regions playing a role in activity are shown in purple. Third conserved region overlapped with motif identified in current study, hence shown in red. B. Three dimensional model of class A beta lactamase (PDC code 1W7F) showing group A specific motif identified in present study (pink) and PROSITE motif (yellow). Dotted surface highlights the active site.

Mapping of the group-specific motifs on the crystal structures showed that motifs identified in this study mapped in close proximity to the active site (Figure 3).

Group A specific motif identified from this study was present on the S-D-N functional element responsible for catalytic activity of class A lactamase [1] whereas PROSITE motif was located on S-X-X-K element. Group A specific PROSITE and motifs identified from this study were present on the opposite boundaries of the active site but are adjacent to each other in three dimensional orientations.

Motifs specific to group C and D identified in this study overlapped with the PROSITE motifs specific to corresponding classes (Table 1). Motifs specific to group C and D were found on S-X-S-K and S-T-F-K regions respectively (Figure 3). In class C, electrostatic interaction between Lys (K) and Ser (S) seems to play role in orientating the active site. Electrostatic interaction between S and K creates a net positive potential in the catalytic site, where the carboxylic group of the beta-lactam is expected to bind. While in class D, active site residue i.e. Ser (S) lie in specific motif (S-T-F-K) identified from this study.

**Table 1:** Comparative location of class specific motifs from PROSITE database with motifs identified in current study.

Motif	Motif (This study)	Prosite Motif	GI number of Reference Sequence
Group A	150-163	88-103	47778058

Group B	117-122	114-133	85374040

Group C	84-92	80-87	28870754

Group D	75-91	73-83	115525541

Group E	69-75	No information	56420626

Group F	91-108	No information	119944862

Motifs specific to group B (corresponding to class B in ambler classification) showed conservation at only protein level (Figure 2). Proteins belonging to group B are highly divergent and heterogeneous [17]. It has been further divided into further subgroups. It is interesting to mention here that both the novel groups identified in this study (Group E and F) has BeTs links to group B. Lack of conservation at DNA level indicates that proteins from this group experience diverse evolutionary forces and may be case for convergent evolution. The location of motifs specific to group B overlapped with the PROSITE motifs specific to corresponding class (Table 1). Motifs specific to group B was found on H-X-H-X-D which is involved in specific interactions with zinc ion (Zn^2+^), required for activity of this class of beta-lactamase [18].

Proteins from group E lack lactamase domain. Motifs specific to group E showed conservation at protein level. Lack of conservation at DNA level and BeTs links to group B indicates that proteins from this group are likely to be highly divergent proteins belonging to group B. Length of proteins belonging to group E is similar to proteins from group B. Both groups E and B showed conserved histidine (H) and aspartic acid (D) at the sequence level.

Motifs from group F showed higher conservation at both protein and DNA level (Figure 2). The proteins from this group lacked lactamase domain. Motifs specific to group F contains conserved G, F, D and E [Table 2].

**Table 2:** Comparison of group-specific motifs identified in this study to the PROSITE motifs.

A)			
**Group**	**Pattern**	**Sensitivity**	**Specificity**

A	S-[DG]-N-x(1,2)-A-[ACGNST]-x(2)-[ILMV]-x(4)-[AGSTV]	70.270	99.048

B	H-x-[DEH]-x-D-H	82.556	100

C	S-x-[AEGQST]-K-x(2,4)-T	80.851	90.297

D	S-T-[FY]-K-[IV]-x(2)-[AST]-[LMV]-[FILM]-[AG]-x-[ADEQS]-x(3)-[ILV]	71.429	97.561

E	D-[IV]-R-x(3)-E	80.435	88.095

F	G-x(2,3)-F-x-[GST]-x-[QST]-D-x(1,2)-E-[IV]-[IL]-[AIL]-x-[GL]-x-[AES]	87.5	100

			
**B)**			

**Class**	**Pattern**	**Sensitivity**	**Specificity**

A	[FY]-x-[LIVMFY]-{E}-S-[TV]-x-K-x(3)-{T}-[AGLM]-{D}-{KA}-[LC]	42.4	97.5

B	[LI]-x-[STN]-[HN]-x-H-[GSTAD]-D-x(2)-G-[GP]-x(7,8)-[GS]	1.8	100

C	[FY]-E-[LIVM]-G-S-[LIVMG]-[SA]-K	7.5	69

D	[PA]-x-S-[ST]-F-K-[LIV]-[PALV]-x-[STA]-[LI]	41.1	100

E	Information not available		

E	Information not available		

A) Group-specific motifs identified in this study. B) Motifs available in PROSITE database for class A, B, C and D lactamases

Motifs found in class E and F were very specific to beta-lactamase, but we were unable to find any lactamase domain because these classes are highly divergent. However, we suspect proteins belonging to these groups as beta-lactamase because group E and F proteins showed similarity to experimentally identified lactamase proteins which we used in initial screening. Proteins from both groups showed Bi-Directional Best Hit (BeTS) relationships with beta-lactamases from other characterized classes e.g. both group E and F has BeTS links with B. The BeTs are generally considered as criterion for defining orthology (functional equivalence).

## Discussion

The present work was carried out with two objectives (i) to evaluate the classification of lactamase genes on a large dataset and (ii) to identify specific motifs/regions which can be further developed to diagnostic primers and probes. The results show that the graph-based classification of beta-lactamase proteins from DLact database corresponds with the classification proposed by Ambler [5], thus there is no need for formulating a new classification. However, identification of two small groups indicates that an update of the existing classification scheme may be required. The identity of proteins from group E and F as lactamases has been defined on the basis of their initial homology with the experimentally characterized lactamases and then later their BeTs relationship with group B lactamases. However, proteins from both these groups do not contain lactamase domain. The proteins are labelled as uncharacterized in various genome databases and annotation sites. It is critical therefore that experiment should be conducted on these groups.

The group-specific motifs identified from this study showed better sensitivity and specificity in comparison to the motifs available in PROSITE. It is likely that the motifs identified using larger dataset have identified stronger consensus regions and suppressed weakly conserved regions. Proximity of these motifs to the class specific active sites indicates that these regions are either structurally of functionally important for the lactamase activity. The sequence logos of regions containing class-specific motifs show that these regions have lesser substitution rates as compared to the other regions in proteins. Thus it is likely that these regions can be used to develop diagnostic probes. We are studying the co-occurrence profiles of motifs in order to identify non-overlapping regions which can be developed into sensitive class-specific primers.

The study has achieved its objectives in terms of evaluating classification of beta-lactamases proposed by Ambler [5] on a large dataset and identifying group-specific motifs.

## Conclusion

The graph-based classification of beta-lactamase proteins from DLact database corresponds with the four group classification proposed by Ambler, thus there is no need for formulating a new classification. However, further characterization of two small groups may require updating the existing classification scheme. Group-specific motifs identified from six groups in this study had high sensitivity (> 70%) and very high specificity (> 90%), as compared to PROSITE motifs, and their proximity to the active site indicates that these motifs represents characteristic group-specific signature of beta-lactamases and can be further developed into better diagnostics and therapeutics.

## Competing interests

The authors declare that they have no competing interests.

## Authors' contributions

RS has made substantial contributions to the analysis and interpretation of data, and drafting the manuscript. AS has participated in the mapping of patterns on the structure. HS has conceptualization of idea and has provided directions for the work.
